# Musca Volitentes

**Published:** 1875-11

**Authors:** John Coston

**Affiliations:** Bowdon, Georgia


### MUSCA VOLITENTES.

BY JOHN COSTON, M. D., BOWDON, GEORGIA.

Permit me to present to you the history of a case under my care, since February last; and to do this properly, it is necessary to give a short sketch of the case previous to the time I took charge of it.

Miss L——— was taken, in February, 1872, with musca volitentes soon after the ground had been covered for sometime with snow. Some few months thereafter, she was placed under the professional care of Dr. H., who treated her for a while to no effect; then under Dr. A., a botanic, whose treatment also failed to cure.

Dr. P., a regular physician, who reports prolapsus uteri, with uterine leucorrhœa, or endo-metritis, then treated her for a time with like results—after which Dr. B , a botanic, who confirms the prolapsus and reports ulcers, treated her, but likewise failed to cure, until, in February last, I was requested to take charge of and treat her case, when I found the following conditions to exist:

Miss L., aged 18 years; phlegmatic temperament; full and plump in the face, but having a rather sallow, whitish color of skin, with a somewhat bloated aspect; strictly confined to bed; tongue and pulse normal; appetite weak; complains of sour stomach, whether she eats much or little; great tightness of chest occasionally—not a fair globus hystericus, but much on that order; deep-seated, aching pain across the sacral and abdominal regions; weight and bearing-down in the pelvis; pains down the thighs; extreme hyperasthesia of surface, especially in the dorsal region; extremely nervous; easily frightened; greatly depressed in spirits; sleeps but little; cannot read, owing to the superorbital pain, induced by any steady use of the eyes; musca volitentes very bad; multitudes of specks and chars before the eyes; no perceptible derangement of lungs, liver or heart, but uterus prolapsed, but no ulcers, as reported by others; but, to my astonishment, I found the bowels completely impacted, and, from the history I got, the bowels have been badly constipated for the last several years, even before the eyes were affected.

With these lights before me, I determined to regulate the bowels the first thing, and, to accomplish this, resort was had to cathartics, and no satisfactory results, until I put her on strychnia, and on belladonna, in accordance with Baxter's theory, that it operates on the longitudinal fibers. After a regular use of it for sometime, it succeeded in breaking up the impaction, when quite a quantity of dry fæcal matter was discharged—as described by the mother—resembling dry oak leaves rubbed up. Soon after this, the sour stomach disappeared, the tightness of the chest better, a regular improvement going on, one symptom after another giving way, until in May, when incidentally I learned she received a fall, while in school in 1867. Upon inquiry of her and her mother, I learned that she fell with the nates astride the edge of a brick, and, for months thereafter, she suffered greatly with pain about the coccyx; and upon examination, I found a disarticulation and dislocation at the sacro-coccycal articulation, the upper end of the coccyx lying on the dorsum of the sacrum, the lower end turned to the left side and pressed inward—the lower end exquisitely tender, pressure thereon giving rise to the abdominal and sacral pains formerly spoken of. Upon this discovery, I applied a blister over the coccyx, and now have it running, by the application of croton oil, and she has not taken a cathartic (which she had to do previously every time the bowels were moved) now, in eight or ten weeks, musca volitentes much better, can now read all day without pain, appetite good, no sour stomach, hyperasthesia almost gone, sleeps well, nervousness much relieved, cheerful, hopeful, and in every way much more comfortable, visits from house to house on foot, or in a buggy, without complaint.

And now the question arises, What connection has this fall with all this train of ailments? Just this: by this displacement of the coccyx, the muscles and ligaments on the anterior surface of the sacrum and coccyx are put on the stretch; thereby unusual pressure is made on the sacral ganglia and on the ganglia imparthus, pressing and benumbing the nerves and deranging their functions, and, through sympathy, one ganglia after another becomes involved, the bowels cease to perform their regular perestatic and vermicular contractions and dilatations, secretion fails, constipation ensues, by continued nervous derangement or debility indigestion is produced, by sympathy the optic nerve becomes involved, musca volitentes is produced, hyperasthesia follows and muscular relaxation supervenes, and hence the muscles and ligaments supporting the uterus relax and allow it to descend, etc. Thus I trace the whole train of ailments to the fall, for you perceive, from the foregoing sketch, that I had traced the constipation back far toward the time of this accident, before I knew of its occurrence.

General treatment: alteratives, tonics and anodynes.

Now, I think I have reached the first cause in the case: will you be so kind as to give me your opinion as to the correctness of my deductions? And any suggestions as to the treatment will be gladly and thankfully received.

[Your logic is good, and, as your patient is improving, your diagnosis and treatment must be correct.—P.]

